# The Application of Ribozymes and DNAzymes in Muscle and Brain

**DOI:** 10.3390/molecules15085460

**Published:** 2010-08-09

**Authors:** Nikolaos P. Mastroyiannopoulos, James B. Uney, Leonidas A. Phylactou

**Affiliations:** Department of Molecular Genetics, Function & Therapy, The Cyprus Institute of Neurology & Genetics. PO Box 23462, Nicosia, Cyprus; Henry Welcome Laboratories for Integrative Neurosciences and Endocrinology, University of Bristol, Whilston street, Bristol, BS13NY, UK

**Keywords:** catalytic nucleic acids, ribozyme, DNAzyme, brain, muscle

## Abstract

The discovery of catalytic nucleic acids (CNAs) has provided scientists with valuable tools for the identification of new therapies for several untreated diseases through down regulation or modulation of endogenous gene expression involved in these ailments. These CNAs aim either towards the elimination or repair of pathological gene expression. Ribozymes, a class of CNAs, can be mostly used to down-regulate (by RNA cleavage) or repair (by RNA *trans*-splicing) unwanted gene expression involved in disease. DNAzymes, derived by *in vitro* selection processes are also able to bind and cleave RNA targets and therefore down-regulate gene expression. The purpose of this review is to present and discuss several applications of ribozymes and DNAzymes in muscle and brain. There are several diseases which affect muscle and brain and catalytic nucleic acids have been used as tools to target specific cellular transcripts involved in these groups of diseases.

## 1. Introduction 

Selective gene silencing by catalytic nucleic acids is a field that has been used with great success for studying natural processes in muscle and brain. Furthermore, these catalytic nucleic acids have been widely used for establishing novel gene therapy approaches for many muscle and brain diseases. In most cases, catalytic nucleic acids aim towards either the elimination or repair of pathological transcripts. Catalytic nucleic acids can be separated into two main categories: ribozymes and DNAzymes. Ribozymes, the most extensively studied of the catalytic nucleic acids, exist in a range of distinct categories of naturally occurring catalytic RNA. These include a series of small ribozymes important for the rolling circle replication of viroid genomes, such as hammerhead and hairpin ribozymes [1,2,3], group I introns [4,5,6], the RNA component of RNase P [7], and hepatitis delta virus ribozyme [8]. Several recent reviews have described and discussed the various catalytic nucleic acids [6,9,10]. The hammerhead, hairpin and hepatitis delta virus (HDV) ribozyme motifs can be characterized by their ability for self-cleavage of a particular phosphodiester bond [9,11]. Hammerhead ribozymes have the ability to suppress gene expression through specific cleavage of RNA molecules [9]. Group I Intron ribozymes can be characterized by their capacity for self-splicing by cleavage and ligation of phosphodiester bonds [6,11]. Group I intron ribozymes can be designed to act in *trans* by recognition and separation of RNA molecules in a sequence specific manner, and ligation of a new RNA sequence to the separated RNA molecules [6]. DNAzymes, by contrast to ribozymes, have not been observed in nature and are derived by *in vitro* selection processes. One of the most characterized DNAzyme is the 10-23 subtype consisting of a cation-dependent catalytic core of 15 deoxyribonucleotides that binds to and cleaves its target RNA between an unpaired purine and paired pyrimidine through a de-esterification reaction [10,12]. The catalytic activity and specificity of both ribozymes and DNAzymes has been extensively characterized *in vitro* and in cell culture systems. A wide range of chemical modifications has allowed the synthesis of oligonucleotides with *in vivo* stability approaching that of most conventional drugs [13].

Beside ribozymes and DNAzymes, advances in molecular genetics have revealed new tools for selective gene silencing. Small interfering RNAs (siRNAs) are single strands of RNA, approximately 20 nucleotides long, which are able to target, cleave and inactivate specific mRNAs [14]. This powerful tool for the down regulation of mRNA levels is widely used for the potential treatment of many diseases [15,16]. The high specificity and suppression of pathogenic RNA by using siRNA technology proves to be of high expectations. Nevertheless, like ribozymes and DNAzymes, siRNA technology shows some limitation on the selectivity, delivery and stability [14]. Efficient delivery and distribution of oligonucleotide compounds both intracellularly and *in vivo* remains a critical challenge for successful transition from the laboratory to the clinic. 

Beside siRNAs, antisense oligonucleotides have been used to inhibit gene expression levels both *in vitro* and *in vivo* [17]. Antisense oligonucleotides usually consist of 18-25 bases long in antisense orientation to the mRNA of interest. Hybridization of the antisense oligo to the target mRNA results in RNAse H cleavage of the mRNA and prevents protein translation thereby blocks gene expression [18]. During the last twenty years, antisense oligonucleotides have been widely used in basic research, genomics and drug discovery. This review will focus on the use of ribozymes and DNAzymes towards the study and potential therapy of muscle and brain diseases.

## 2. The Use of Ribozymes and DNAzymes in Muscle 

**Figure 1:**
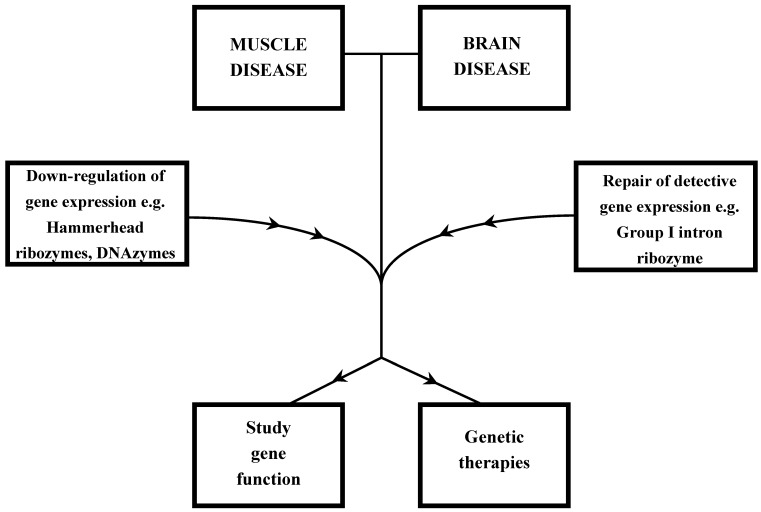
Strategies for the study or therapy of muscle and brain diseases by using ribozymes and DNAzymes.

Ribozymes and DNAzymes have been widely used towards the better understanding and therapy of various skeletal and smooth muscle disorders. Myotonic dystrophy type I, myotonia congenita and neuromuscular junction abnormalities, restenosis of coronary arteries and hypertension are some of the disorders that scientists introduced the use of ribozymes and DNAzymes (Figure 1). 

In this review, we will focus on different strategies implicating the function of ribozymes and DNAzymes in the pathomechanisms and therapy of these disorders (Table 1). 

The vascular smooth muscle cell in mature animals is a highly specialized cell whose principal function is contraction and regulation of blood vessel tone-diameter, blood pressure, and blood flow distribution. However, abnormal environmental signals can lead to adverse phenotypic switching and acquisition of characteristics of smooth muscle cell that can contribute to the development and/or progression of vascular disease [19,20]. Several attempts have been reported which utilised catalytic nucleic acids in smooth muscle cells. References to several reports which demonstrate the application and usefulness of ribozymes and DNAzymes in smooth muscle cells are presented below.

Angiotensin II (Ang II) plays an important role in the development of hypertension and atherosclerosis by inducing vascular smooth muscle cell growth and synthesis of aldosterone [21,22]. Activation of Leukocyte-type 12-lipoxygenase (12-LO) has been proposed to be an important mechanism for AngII by inducing hypertrophy of vascular smooth muscle cells. This finding prompted scientists to design a chimeric RNA hammerhead ribozyme against the first GUC sequence at nucleotide 7 of porcine leukocyte 12-LO mRNA. The ribozyme was transfected into porcine aortic vascular smooth muscle cells, causing a significant decrease of endogenous porcine leukocyte-type 12-LO mRNA and protein levels [23]. Downregulation of 12-LO levels, have the potential to protect vascular smooth muscle cells from hypertrophy [24]. The results from this study indicated the feasibility of using new ribozyme technology to study the specific effects of a gene pathway in vascular disease and the potential therapies.

Proliferation of injured smooth muscle cells contributes to the reocclusion or restenosis of coronary arteries that often occurs following angioplasty procedures. Coronary angioplasty is an effective procedure used in order to open occluded vessels. However, in spite of a number of technical improvements in the procedure, post-operative occlusion of arteries, or restenosis, still occurs. It is widely believed that by preventing the injury-induced activation and proliferation of medial smooth muscle cells after angioplasty, intiminal thickening and restenosis could be prevented [25]. In an attempt to inhibit smooth muscle cell proliferation, Jarvis *et al*. described the activity of several ribozymes targeting c-myb mRNA. Hammerhead ribozymes were capable of cleaving c-myb RNA and as a result inhibit smooth muscle cell proliferation [26]. This finding indicated that hammerhead ribozymes have the potential to inhibit the hyperproliferation of smooth muscle cells that occurs in many patients after coronary angioplasty. In another attempt to prevent restenosis in coronary arteries, Grassi *et al*. explored the use of hammerhead ribozymes as tools to knock down specific activators of cell proliferation. They designed two specific hammerhead ribozymes to inhibit the mRNA levels of cyclin E and E2F1, two potent activators of cell proliferation which cooperate to promote the G1 to S phase transition [27,28]. After transfecting these hammerhead ribozymes within coronary smooth muscle cells, mRNA and protein levels of cyclin E and E2F1 were significantly reduced. Furthermore, the coronary smooth muscle cell growth was dramatically shut down and almost completely prevented when the two hammerhead ribozymes were administered together [29]. 

Ribozymes and DNAzymes have been also used as tools to target defects in neuromuscular diseases. Myotonic Dystrophy type 1 (DM1) is a degenerative neuromuscular disease characterised by a large CTG repeat expansion situated in the 3’ UTR of the DMPK gene. The mutant DMPK 3’ UTR RNA containing the CUG expansion accumulate to form RNA foci in the nucleus of DM1 cells [30,31]. These RNA foci interact with nuclear RNA binding proteins preventing their export from the nucleus [32,33]. The fact that most of the disease pathomechanisms involve mutant RNA foci and their interactions to various binding proteins, prompted researchers to create specific ribozymes for the destruction of the mutant RNA. Langlois *et al*. identified most of the accessible ribozyme target sites in the 3’ UTR of the DMPK mRNA and designed a hammerhead ribozyme to cut the most accessible site. The use of these hammerhead ribozyme significantly reduced the number of mutant DMPK mRNA-containing nuclear foci in human DM1 myoblasts. The reduction of mutant DMPK mRNA and nuclear foci had as an effect the partial restoration of insulin receptor isoform B expression in DM1 myoblasts [34]. 

Another approach was the use of specific ribozymes for the repair of the expanded repeat. Phylactou *et al*. designed a group I intron ribozyme in order to modify the CUG repeat expansion at the 3’ UTR of the human DMPK transcripts. This group I intron ribozyme showed to be capable of ligating a small mRNA fragment, contained within the ribozyme, to a simple DMPK-target RNA *in vitro*. It also modified a larger target transcript, leading to replacement of twelve repeats with five repeats, both *in vitro* and in mammalian cells [35]. 

The mutant canine skeletal muscle chloride channel (cClC-1) mRNA transcript that causes the inherited disorder myotonia congenita, prompted scientists to further investigate the feasibility of RNA repair using specific ribozymes. Rogers *et al*. designed a modified Tetrahymena ribozyme to mediate *trans*-splicing repair of the mutant canine skeletal muscle chloride channel (cClC-1) mRNA transcripts. The ribozyme was able to target the mutant mRNA and replace the mutant containing 3’ portion by trans-splicing the corresponding wild type sequence. Furthermore, when the chloride channel function was examined in single cells, a wide range of electrophysiological activity was observed, with 18% of cells exhibiting significant functional restoration and some cells exhibiting complete rescue of the biophysical phenotype [36].

The muscle acetylcholine receptor (AChR) is expressed at the neuromuscular junction, and plays the principal role in nerve to muscle signal transmission. A number of mutations have been characterised in the AChR -subunit gene which affect receptor function and give rise to slow channel congenital myasthenic syndrome [37,38]. Abdelgany *et al*. designed hammerhead ribozymes in order to target RNA transcripts from four different slow channel congenital myasthenic syndrome mutations. These hammerhead ribozymes were able to efficiently discriminate between mutant and wild type RNA transcripts that differ only by a single nucleotide substitution [39]. Furthermore, the ability of DNAzymes to cause allele-specific cleavage in transcripts where the mutation creates a putative cleavage site or full DNAzyme:target binding was tested. Alelle-specific cleavage was demonstrated in both cases under simulated physiological conditions [40].

## 3. The Use of Ribozymes and DNAzymes in Brain 

The use of ribozyme and DNAzyme strategies were also introduced in various brain disorders (Figure 1). Huntington’s, Alzheimer’s and Parkinson’s are some of brain diseases that ribozymes and DNAzymes were used for their study and therapy (Table 1). Huntington’s disease (HD) is a progressive brain disorder that causes uncontrolled movements, emotional problems, and loss of thinking ability. The underlying cause of Huntington’s disease is the inheritance of a copy of the gene encoding huntingtin with an expanded polyglutamine-encoding CAG repeat located within the 5′ end of the coding region [41]. The mutant huntingtin protein is expressed during development through adulthood, causes neuronal dysfunction, and ultimately cell death of neurons in the striatum. The neuropathology is present to a varying extent in other regions of the brain [41]. Yen *et al*. demonstrated the first effective destruction of the mutant huntingtin mRNA using a specific DNAzyme that was able to cleave the mutant huntingtin mRNA in a sequence-specific manner, that lead to significant reduction of mutant huntingtin protein expression in mammalian cells [42]. Therefore, with the reduction of the mutant huntingtin, several pathways associated with huntingtin were altered and less cellular toxicity was observed. Cellular and animal models showing significantly reduced huntingtin levels, reduce the severity of the disease, suggesting that HD might be reversible [43,44]. 

Catalytic nucleic acids have been also used in Alzheimer’s disease (AD). AD is a neurodegenerative disorder involving the deposition of senile plaques in the brain. The plaques consist of aggregates of a 4 kDa Aβ-amyloid peptide (Aβ). The peptide is produced by sequential cleavage of the amyloid precursor protein (APP) by β- and γ-secretases [45,46]. The level of β-amyloid peptide, one of the major components of toxic amyloid plaques, depends directly on the hydrolytic activity of β-secretase. Nawrot *et al*. designed RNA-cleaving ribozymes in order to control the expression of β-secretase. The scientific findings demonstrated that these ribozymes were able to significantly inhibit β-secretase gene expression at both the mRNA and protein levels [46]. Furthermore, the inhibition of β-site APP cleaving enzyme influenced the total population of β-amyloid peptide and as a result, it can be considered as a molecular tool for the anti-amyloid treatment of AD disease. 

In another study, Kanamori *et al*. showed that an mRNA transcribed in dihydrolipoamide succinyltransferase (DLST) gene was significantly lower in the brain of AD patients compared to the controls [47]. The truncated gene product (designated MIRTD) localized to the intermembrane space of mitochondria. A dimeric hammerhead ribozyme (maxizyme) was targeted against the MIRTD transcript. SH-SY5Y neuroblastoma cells transfected with maxizyme constructs reduced MIRTD expression. The ribozyme-expressing cells had a greater sensitivity to hydrogen peroxide and a significantly reduced rate of respiration, implicating MIRTD in the assembly of the cytochrome C oxidase complex, whose defect has been a candidate of the causes of AD [47,48].

Data from another AD study implicated the double-stranded RNA dependent protein kinase (PKR) in disease progression [49]. A library of hammerhead ribozyme genes was employed to identify genes involved in tunicamycin-induced apoptosis of SK-N-SH cells. Results from screening the ribozyme library led to the identification of PKR, which subsequently was found to be elevated in the brains of AD patients.

Parkinson’s disease (PD) is another neurodegenerative disorder which is characterized by selective degeneration of substantia nigra dopamigenic neurons and the presence of abnormal cytoplasmic aggregates of a-synuclein [50]. Scientific reports showed that a-synuclein overexpression creates toxic effects to dopaminergic neurons [51,52]. By contrast, down-regulation of a-synuclein is effective for inhibition of progressive pathogenesis of PD. Hayashita-Kinoh *et al*. used adeno-associated virus (AAV) vector delivery of a-synuclein ribozyme in order to test its silencing effect on degenerating nigrostriatal neurons in a PD animal model, expressing the 1-methyl-4-phenylpyridinium (MPP+) [53]. MPP+ is a neurotoxic compound found in dopaminergic neurons and is responsible for up-regulation of a-synuclein [54]. Hayashita-Kinoh *et al*. designed a ribozyme against human a-synuclein gene expression and constructed a ribozyme-expressing rAAV vector (designated rAAV-SynRz). Co-transfection of rAAV-SynRz and rAAV-a-synuclein into HEK293 cells resulted in down-regulation of a-synuclein protein expression *in vitro*. rAAV-SynRz was then injected into the substantia nigra of MPP+-treated rats. Cell counts of tyrosine hydroxylase (TH)-positive neurons in the substantia nigra revealed that rAAV-SynRz significantly protected TH-positive cells against apoptotic death, compared with untransfected mice or those which were injected with a control reporter vector [53]. These results showed that the use of rAAV-SynRz allowed the survival of higher number of TH-positive neurons in substantia nigra in the MPP+ model. Furthermore, these results suggest that down-regulation of a-synuclein expression could be a potentially suitable route for gene therapy of PD. 

Glioblastomas are the most frequent malignant brain tumors, which can progress from lower grade gliomas or arise *de novo*. Glioblastomas are lethal and nearly all patients die within one year, despite treatment [55]. Ribozymes and DNAzymes have been exploited to tackle glioblastoma through RNA-targeted strategies [55,56].

The secreted growth factor pleiotrophin (PTN), also called heparin binding growth-associated molecule (HB-GAM), heparin affin regulatory peptide (HARP), heparin-binding growth factor 8, heparin-binding neurotrophic factor (HBNF), or osteoblast-specific protein-1 (OSF-1) is a 15.3-kDa developmentally regulated cytokine, which shows very limited expression in normal adult tissues, but is markedly upregulated in various primary human tumors and tumor cell lines [55,57]. Scientists have tested the hypothesis that the growth of human glioblastomas can be controlled by regulating PTN expression. Grzelinski *et al*. showed in two cell lines, U87 and T98G, that stable ribozyme-targeting leads to a robust reduction of PTN mRNA and protein levels. Further investigations showed that the reduction of PTN mRNA and protein levels results in decreased migration and colony formation in both glioblastoma cell lines [55]. When the same strategy was followed in a mouse model, angiogenesis and tumor growth were markedly reduced upon PTN depletion, paralleled by decreased PTN serum levels [55]. In addition, PTN was found to signal through anaplastic lymphoma kinase (ALK) [58]. ALK was found to be upregulated in glioplastoma compared to normal brain and when bound to PTN growth stimulatory and antiapoptotic effects are mediated [59]. Grzelinski *et al*. used a subcutaneous tumor xenograft model in order to transfect specific ribozymes against both PTN and ALK, this had as a result the abolishment of tumor growth [60]. 

Clinical and experimental evidence showed that the metastasis of malignant cells from a localized tumor is directly related to the number of microvessels in the primary tumor. Tumor angiogenesis is thought to be mediated by tumour-cell-derived growth factors. PTN can induce the release of active proteolytic enzymes from endothelial cells and induce tube formation of endothelial cells *in vitro*. It may well be able to serve as a tumor angiogenesis factor [61]. To test the above hypothesis, Czubayko *et al*. transfected human melanoma cells expressing high PTN levels, found to be able to metastasize from subcutaneous tumors to the lungs of experimental animals, with ribozymes in order to reduce PTN expression levels. The reduction of PTN mRNA levels showed not to affect the growth of the melanoma cells *in vitro.* In nude mice, however, tumor growth and angiogenesis were decreased. Moreover, apoptosis in the tumors was increased and the metastatic spread of tumors from the subcutaneous site to the lungs was prevented [62]. 

Finally, DNAzymes were used against the Japanese encephalitis virus (JEV). JEV is an arthropod-borne flavivirus with a single-stranded RNA genome containing non-coding regions at its 5’ and 3’-ends [63]. Non-coding regions have flavivirus-conserved sequences that are important for virus replication. As previously described, DNAzymes are single-stranded oligodeoxynucleotides (ODNs) with Mg^2+^-dependent enzymatic activity capable of cleaving single-stranded RNA at specific sites under simulated physiological conditions [64]. These DNAzymes were able to specifically target the NCRs and inhibit JEV replication in culture cells and infected animals [65]. Appaiahgari *et al*. showed that intra-cerebral administration of a poly-(G)(10)-tethered, phosphorothioated DNAzyme in JEV-infected mice led to more than 99.99% inhibition of virus replication in brain, resulting in a dose-dependent extended lifespan or complete recovery of the infected animals [65].

## 4. Conclusions 

To date, several different therapeutic attempts have been described as potential treatment approaches for many muscle and brain diseases. The use of catalytic nucleic acids and more specifically of ribozymes and DNAzymes against these diseases has shown some promise. Several approaches have already been described in the literature which reveal the potential of ribozymes and DNAzymes as tools for the study or therapy in muscle and brain diseases. The sequence-specific action which both of these categories of catalytic nucleic acids have against target RNA makes them flexible agents for the elimination or repair of pathological gene expression in muscle and brain cells. Particularly the trans-splicing ribozyme has the unique feature to repair mutant transcripts which are implicated in both groups of diseases. Naturally, more effort should be dedicated, particularly in the area of delivery of nucleic acids so that the effect of both ribozymes and DNAzymes is more efficient and specific. 

## Figures and Tables

**Table 1:** Ribozymes and DNAzymes used for the elimination or repair of pathogenic gene expression involved in different muscle and brain diseases.

Ribozyme-DNAzyme	Disease	Gene-Gene target	Reference
Hammerhead ribozyme	Vascular smooth muscle cell hypertrophy-Restenosis	Leukocyte-type 12-lipoxygenase (12-LO)	Gu, J.L., *et.al*. *Circ. Res*. 1995, *77*, 14-20
Hammerhead ribozyme	Vascular smooth muscle cell hypertrophy-Restenosis	Proto-oncogene c-*myb*	Jarvis, T.C., *et al*. *RNA*, 1996, *2*, 419-428.
Hammerhead ribozyme	Vascular smooth muscle cell hypertrophy-Restenosis	Cyclin E and Cyclin E2F1	Grassi, G., *et al*., J Gene Med, 2005. 7(9): p. 1223-1234.
Hammerhead ribozyme	Myotonic Dystrophy type 1 (DM1)	Mutant DMPK 3’ UTR	Langlois, M.A., *et al*., Mol Ther, 2003. 7(5 Pt 1): p. 670-680.
Group I intron ribozyme	Myotonic Dystrophy type 1 (DM1)	Mutant DMPK 3’ UTR	Phylactou, L.A., *et al*., Nat Genet, 1998. 18(4): p. 378-381.
Hammerhead ribozyme	Slow channel congenital myasthenic syndrome	Acetylcholine receptor (AchR)	Abdelgany, A., *et al*., J RNAi Gene Silencing, 2005. 1(1): p. 26-31.
DNAzyme	Slow channel congenital myasthenic syndrome	Acetylcholine receptor (AchR)	Abdelgany, A., *et al*., J RNAi Gene Silencing, 2005. 1(1): p. 32-37.
DNAzyme	Huntington’s	Huntingtin (HTT)	Yen, L., *et al*., Ann Neurol, 1999. 46(3): p. 366-373.
Hammerhead ribozyme	Alzheimer’s	β-Secretase	Nawrot, B., *et al.*, Eur J Biochem, 2003. 270(19): p. 3962-3970.
Hammerhead ribozyme	Alzheimer’s	Dihydrolipoamide succinyltransferase (DLST)	Kanamori, T., *et al*., EMBO J, 2003. 22(12): p. 2913-2923.
Hammerhead ribozyme	Parkinson’s	a-Synuclein	Hayashita-Kinoh, H., *et al*., Biochem Biophys Res Commun, 2006. 341(4): p. 1088-1095.
Hammerhead ribozyme	Glioblastomas	Pleiotrophin (PTN)	Grzelinski, M., *et al*., Int J Cancer, 2005. 117(6): p. 942-951.
Hammerhead ribozyme	Glioblastomas	Pleiotrophin (PTN) and anaplastic lymphoma kinase (ALK)	Grzelinski, M., *et al*., Neoplasia, 2009. 11(2): p. 145-156.
Hammerhead ribozyme	Encephalitis	Japanese encephalitis virus (JEV)	Appaiahgari, M.B. and S. Vrati. Mol Ther, 2007. 15(9): p. 1593-1599.
